# Intraoperative Platelet Rich Plasma Usage in Total Knee Arthroplasty: Does It Help?

**DOI:** 10.1155/2013/740173

**Published:** 2013-07-28

**Authors:** Thomas B. Pace, Jonathan L. Foret, M. Jason Palmer, Stephanie L. Tanner, Rebecca G. Snider

**Affiliations:** Department of Orthopaedics, Greenville Health System, Greenville, SC 29605, USA

## Abstract

Autologous platelet rich plasma preparations, commonly referred to as platelet gel, have been reported to have benefits when used in total knee replacement of less blood loss and better motion, with few reported complications. This retrospective review of 268 consecutive primary total knee arthroplasty cases compares postsurgical range of motion at 2, 8, and 12 weeks, knee manipulation rates, change in hemoglobin, and complications between one group receiving a platelet gel preparation (135 cases), and an equivalent group receiving no platelet gel preparation (133 control cases). No difference was found between groups in manipulation rates, knee range of motion, or changes in hemoglobin (*P* > 0.05). The use of platelet gel in this study did not have a significant effect on hemoglobin at 72 hours postoperatively, knee range of motion, or manipulation rates up to 3 months post-op in this patient cohort.

## 1. Introduction

A wide range of benefits have been reported with autologous plasma preparations administered into the surgical wounds with few reported problems [1–3]. Autologous platelet gel is a platelet rich plasma substance that is derived from a patient's blood. When used in total knee arthroplasty, 55 cc of blood is taken from the patient immediately prior to surgery, centrifuged to separate the blood components and allow extraction of the platelet rich and platelet poor plasma. This combination contains a mixture of plasma, fibrin, and platelets as well as growth factors, clotting factors, and vascular endothelial and fibroblast stimulants [4]. The mixture is stored in a syringe and mixed with bovine thrombin during its application to the surgical wound. The clinical effects of autologous platelet gel when used in total knee arthroplasty have been evaluated by several different authors who found varying degrees of benefit from its use. Some reported benefits include improved knee range of motion, decreased manipulation rates, decreased blood loss, and decreased hospital length of stay [1–3].

This review will compare the effect of platelet gel usage in total knee replacement surgery with respect to surgical blood loss, postoperative complications, range of motion, and knee function score. 

## 2. Materials and Methods 

Institutional Review Board approval was obtained for this study. There is no conflict of interests with any of the authors. A retrospective review was conducted of 268 consecutive primary total knee replacements in 262 patients all performed by the senior author between June 2006 and October 2008. The first 135 surgeries received platelet gel and the subsequent 133 surgeries did not. Demographics are shown in Table 1. 

Six patients underwent bilateral knee replacements, but none were done simultaneously. For 12 consecutive months, platelet gel was used on all patients (135 surgeries). This group was compared to the remaining patients (133) in the study time period that received no platelet gel but only thrombin spray and served as the control group.

A combination of femoral nerve block and spinal anesthesia was used in all patients. All cases had an upper thigh tourniquet inflated to 300 mm/Hg after exsanguination and released once the deep fascial layers were closed. All components were cemented, and there was no difference between the groups regarding preop range of motion, diagnosis, type of implant (Zimmer NexGen), patella resurfacing, surgical approach, or technique. For patients with smaller diameter thighs, a subvastus medialis approach (SVM) was performed and for others a medial parapatella approach (MPP). Posterior cruciate ligament (PCL) sacrifice and lateral retinacula releases were performed based on soft-tissue balancing, patella tracking assessments and were applied equally to all patients.

### 2.1. Platelet Gel Treatment Group

Fifty-five milliliters of whole blood was obtained from patients in the presurgical preparation area. After the blood was centrifuged, the platelet rich plasma was removed, mixed with a portion of the platelet poor plasma, and placed on the surgical table for use prior to wound closure. The platelet gel preparation and a syringe of bovine thrombin were placed separately into the application device. Just prior to wound closure, the platelet gel and thrombin mixture were sprayed onto the implants and the deep and superficial tissues. 

### 2.2. Nonplatelet Gel (Control) Group

Because bovine thrombin is an additive applied with the platelet gel substance, and it alone could have had an effect, the nonplatelet gel group, a 5 cc thrombin spray was used without any platelet gel and was administered to the deep and superficial tissues after implants were in place and prior to wound closure in the same manner as noted previously. This was deemed the control group as it had all properties of the platelet gel group including the additional clotting influence of the added thrombin, but the absence of the processed platelet rich plasma component.

Blood loss after surgery was evaluated by comparing pre- and postsurgical hemoglobin levels for each patient. No patient donated blood preoperatively or received erythropoietin. Hemovac drains were not used postoperatively. All patients received a short course of low molecular weight heparin (Enoxaparin, Sanofi-Aventis, Paris, France) and as well oral warfarin for thromboprophylaxis while hospitalized. Venous thrombosis event (VTE) prophylaxis was 5 mg oral warfarin daily beginning the day of surgery with low molecular weight heparin injections for post-op days 2 and 3 followed by a 4 week duration of 2 mg per day minifixed dose oral warfarin regimen [5] based on individual patient VTE risk assessment. For the patient without higher VTE or bleeding risk assessment, once hospital International Normalized Ratio (INR) levels reached 1.2 or higher, oral warfarin was reduced to 2 mg daily, and postdischarge INR monitoring was not done unless signs or symptoms of bleeding occurred [6]. For higher risk VTE patients, higher dose monitored oral warfarin was used (INR target = 2.0). For patients whose postsurgical hemoglobin levels dropped to 8.0–8.5 mg/dL or lower, transfusion was considered and ensued unless patent request or other medical conditions prohibited transfusion. Physical therapy was standardized following the hospital's total joint rehabilitation protocols and included partial weight bearing postoperative day one and criteria for discharge including independent ambulation, performance of activities of daily living, and passive knee flexion of 80–90 degrees. A continuous passive motion machine was not used after the primary surgery. The same therapy team performed home rehabilitation for two weeks; this was followed by outpatient therapy. A range of motion assessment was performed by the operative surgeon at all follow-up appointments and knee flexion was recorded. Patients who failed to obtain 70 degrees of flexion at 2 weeks were treated with anti-inflammatory medications and diuretics. Formal therapy was held and patients worked on knee flexion exercises at home. Radiographic assessment of the surgical knee was performed at the two, or six week follow-up office visit.

The decision to manipulate was made in the 8- to 12-week period once it was demonstrated that progress in therapy was no longer improving motion, and the patient had less than 90 degrees of flexion. Manipulations were performed in the operating room under general or spinal anesthesia. After manipulation, patients used continuous passive motion machines at home six hours a day for a total of two weeks. Patients were seen daily by home therapy and had a follow-up visit with the surgeon two weeks after manipulation.

Preoperative Knee Society Scores (KSS) [7] were compared to the KSS from the most recent postoperative clinical assessment. Blood loss was evaluated by comparing the change in hemoglobin values (pre-op—lowest recorded post-op) between groups. 

Complications were recorded including manipulations, infections, and wound hematomas requiring surgical evacuation. The decision to evacuate a postoperative wound hematoma was based on continued wound drainage at 10 days. 

Descriptive statistics were performed to analyze the results, including mean, minimum and maximum values. Continuous data was analyzed with Student's *t*-test. Categorical data was compared using Fishers Exact or Chi-square test for proportions. Statistical significance was assigned to *P* value less than 0.05.

## 3. Results 

No differences were found in the patient demographics between the groups (Table 1). 

Preoperative KSS were similar between the groups. The average follow-up time for patients in the nonplatelet gel group was 13 months (range 0.6–32.7 months). The average follow-up time in the platelet gel group was 14.9 months (range 0.6–29.1). 

Analysis of surgical approach and technique yielded no statistical difference (Table 2).

The average change in hemoglobin (preop Hgb—lowest postop Hgb) was similar between the groups (Table 3).

At least 110 degrees of knee flexion was obtained in all patients during the manipulation. There was no difference in manipulation rate between groups (*P* = 1) (Table 4). 

Average knee flexion at 2 weeks, 8 weeks, 12 weeks, and latest follow-up was not statistically different between groups (*P* = 0.33, *P* = 0.52, *P* = 0.91, *P* = 0.16) (Table 5). 

There were more post-operative hematomas requiring evacuation and three patients with infection in the nonplatelet gel group though these differences were not statistically significant (Table 6). The trend is notable.

Other complications included one stroke, one patella fracture, and one symptomatic deep vein thrombosis, all in the nonplatelet gel group (Table 6). There were no symptomatic PE or suspected fatal PEs.

All patients were discharged home on the 3rd or 4th post-operative day based on physical therapy assessments of independence. There was no difference in the hospital stay rates of either group.

## 4. Discussion 

Platelet gel has been studied in a variety of clinical settings including the treatment of burn injuries, spine surgery, lower extremity nonunions, sports medicine, and arthroplasty with varying results [1–3, 8–10]. Specifically, in total knee replacement patients, platelet gel has been reported to decrease blood loss, pain medication requirements, length of hospital stay, and rate of knee manipulation and improve postoperative knee range of motion [1–3]. The increase in motion referred only to flexion 72 hours after surgery. If there is an accelerated proliferation of the fibroblasts to promote faster wound healing and lower infection risk, one could suspect there might be an associated higher risk of postoperative arthrofibrosis following total knee replacement with platelet gel secondary to accelerated scar tissue formation. The present review compares the 2, 8, and 12 week post-op range of motion and knee manipulation rate as a measure of arthrofibrosis following primary total knee replacement in patients receiving intraoperative platelet gel with a group of primary total knee replacements receiving no platelet gel. No difference was found.

The drop in hemoglobin was not significantly different between the two groups in this study. One of the reported benefits of platelet gel is an improvement in postoperative knee range of motion. However, the reported data measured only knee motion up to 72 hours postoperatively. The data from this study showed no difference in the postoperative range of motion at follow-up at 2, 8, and 12 weeks. No difference between the groups with regard to manipulation rate was demonstrated in this study.

The potential for autologous platelet gel to provide a benefit by decreasing infections has been examined by several authors. It has been established that platelets play a role in antimicrobial defense, yet the exact role that platelets play is complex and not completely understood [11, 12]. The high concentration of leukocytes in the platelet gel may contribute to its reported antimicrobial properties against some bacterial species [13]. In this study two patients had infections, both in the nonplatelet gel group, yet the numbers were such that no statistical difference was found between the groups with regard to infection rate.

More patients in the nonplatelet gel group had a wound hematoma requiring surgical evacuation compared to the platelet gel group. In the nonplatelet gel group, three had culture positive wound infections; one had a traumatic fall prior to hematoma evacuation, and two others had documented excess anticoagulation parameters on warfarin therapy. Thus, it is not possible to determine the role of platelet gel in decreasing the number of wound hematomas that required surgical drainage based on this data. The use of thrombin in the nonplatelet gel group was done to further isolate the contributions of PRP on wound healing and blood loss as thrombin is an added agent mixed with the platelet gel and it alone has a known hemostasis effect on surgical blood loss. While its use in both groups did not allow a virgin control group, its usage did allow an assessment of PRP as an isolated agent and from the data gathered in this study, that effect on motion, blood loss, and complications involving total knee arthroplasty did not reach significant levels in any group assessed. 

## 5. Conclusion 

The use of PRP in orthopaedic surgeries has increased in popularity over the past decade. Hospitals are becoming more cost conscious and are requesting clearly defined patient benefits from using additional surgical adjuncts that may offer claims to justify their added expense. This report comparing 238 consecutive total knee surgeries with and without PRP does not show a clear patient benefit from the use of this product with regard to hospital length of stay, blood loss, infection, or improved knee motion. 

## Figures and Tables

**Table 1:** Patient demographics.

	Platelet gel (135)	Nonplatelet gel (133)	*P* value
Average age (range)	67.5 yrs (48–92)	68.7 yrs (38–93)	0.33
Sex	49 M 86 F	43 M 90 F	0.58
Avg Preop KSS (range)	79.3 (68–88)	78.9 (61–86)	0.47
Surgical side	R 83 L 52	R 69 L 64	0.14
Ave Preop Hgb (range)	13.6 (10.1–17.1)	13.6 (10.3–18)	0.76

**Table 2:** Surgical approach and technique.

	Platelet gel	Nonplatelet gel	*P* value
Surgical approach	SVM 41 MPP 94	SVM 35 MPP 98	0.55
PCL sacrificed	50	46	0.77
Patella resurfaced	13	6	0.16
Implant type (Zimmer/NexGen)	135	133	1
Lateral release	20	26	0.42
Avg. tourniquet time (range)	41 min (32–93 min)	41 min (29–74 min)	0.80

**Table 3:** Blood loss evaluation.

	Platelet gel	Nonplatelet gel	*P* value
Preop Hgb (Avg)	13.6	13.6	0.76
Discharge Hgb (Avg)	10.7	10.7	0.85
Lowest recorded Hgb (Avg)	10.3	10.4	0.50
Change in Hgb: preop-lowest (Avg)	−3.3	−3.2	0.25
# Pts lowest Hgb = or < 8.5	13	15	0.81

**Table 4:** Manipulations.

	Platelet gel	Nonplatelet gel
Manipulations	8	6
Avg flexion at manipulation (range)	120 degrees (110–130)	120 degrees (110–125)
Flexion at last f/u (of those manipulated)	110 degrees (80–130)	110 degrees (95–130)

**Table 5:** Range of motion.

	Platelet gel	Nonplatelet gel	*P* value
Avg Postop KSS	99.1	98.9	0.44
Avg Flexion at 2 wks	92.8 degrees	94.1 degrees	0.33
% patients achieving >115 degrees flexion at 8 wks	73.3%	76%	0.52
% patients achieving >115 degrees flexion at 12 wks	80.7%	77.4%	0.61
Avg Flexion at last f/up (range)	119 degrees (80–130)	117 degrees (92–130)	0.16

**Table 6:** Complications.

	Platelet gel	Nonplatelet gel	*P* value
Hematoma requiring surgical evacuation	0	5	0.06
Infection	0	3	0.24
Patella Fracture	0	1	1
Arthrofibrosis	8	6	0.81
Symptomatic DVT/PE	0	1 DVT	1
CVA	0	1	1
